# CD38 Deficiency Promotes Inflammatory Response through Activating Sirt1/NF-*κ*B-Mediated Inhibition of TLR2 Expression in Macrophages

**DOI:** 10.1155/2018/8736949

**Published:** 2018-05-27

**Authors:** Yisong Qian, Chuqiao Chen, Leliang Ma, Ziwei Wang, Ling-Fang Wang, Li Zuo, Yaqin Yang, Xiang Huang, Meixiu Jiang, Xiaolei Wang, Huidong Shi, Mingui Fu, Ke-Yu Deng, Hong-Bo Xin

**Affiliations:** ^1^Institute of Translational Medicine, Nanchang University, Nanchang 330031, China; ^2^Georgia Cancer Center, Augusta University, Augusta, GA 30912, USA; ^3^Department of Basic Medical Science, Shock/Trauma Research Center, School of Medicine, University of Missouri-Kansas City, Kansas City, MO 64108, USA

## Abstract

CD38 was first identified as a lymphocyte-specific antigen and then has been found to be widely expressed in a variety of cell types. The functions of CD38 are involved in numerous biological processes including immune responses. Here, we showed the downregulations of both TLR2 mRNA and protein in macrophages from CD38^−/−^ mice and in CD38 knockdown RAW264.7 cells. Several NF-*κ*B-binding motifs in the promoter region of the TLR2 gene were identified by the bioinformatics analysis and were confirmed by the luciferase activity assay with the different truncated TLR2 promoters. CD38 deficiency resulted in the reduction of NF-*κ*B p65 and acetyl-NF-*κ*B p65 (Ac-p65) levels as determined by Western blot. The expression of Sirt1 did not change, but an increased activity of Sirt1 was observed in CD38-deficient macrophages. Inhibition of the Sirt1/NF-*κ*B signaling pathway resulted in downregulation of TLR2 expression in RAW264.7 cells. However, re-expression of CD38 in the knockdown clones reversed the effect on Sirt1/NF-*κ*B/TLR2 signaling, which is NAD-dependent. Moreover, the inflammatory cytokines including G-CSF, IL-1alpha, IL-6, MCP-1, MIP-1alpha, and RANTES were increased in CD38 knockdown RAW264.7 cells. Taken together, our data demonstrated that CD38 deficiency enhances inflammatory response in macrophages, and the mechanism may be partly associated with increased Sirt1 activity, which promoted NF-*κ*B deacetylation and then inhibited expression of the TLR2 gene. Obviously, our study may provide an insight into the molecular mechanisms in CD38-mediated inflammation.

## 1. Introduction

CD38 is a type II membrane-bound glycoprotein, which is conserved in the evolutionary process and highly expressed in the immune system [1, 2]. CD38 has both ADP-ribosyl cyclase and cADPR hydrolase activities and is capable of cleaving nicotinamide adenine dinucleotide (NAD) to cyclic ADP ribose (cADPR) which is a trigger for intracellular Ca^2+^ release and hydrolyzing cADPR to ADPR, respectively [3]. As a major NADase in mammalian cells, CD38 is a crucial regulator of NAD-dependent deacetylase such as SIRT1 which modulates aging and energy metabolism [4]. In addition, CD38 is an NAADP synthase required for NAADP-mediated Ca^2+^ release from lysosomal stores [5].

The functions of CD38 are involved in numerous biological processes including cell proliferation [6], muscle contraction [7], hormone secretion [8], and inflammatory response [9]. Emerging evidence indicates that CD38 and its downstream NAD/Sirt1 signaling are key regulators in cardiovascular disease and metabolic syndrome. It has been reported that a defection of the CD38/NAADP signaling pathway leads to lysosomal cholesterol accumulation in macrophages and results in coronary atherosclerosis in CD38^−/−^ mice [10]. Our previous work demonstrated that CD38 deficiency protects against obesity, hypertrophy, and myocardial ischemia/reperfusion injury through activating Sirt1 [11–13]. However, the detailed mechanisms of CD38 in the regulation of inflammation and the role of macrophage in CD38-mediated physiological and pathological process remain unknown.

Toll-like receptors (TLRs) are a class of proteins, known as the sensor of pathogen-associated molecular patterns (PAMPs), that play crucial roles in the innate immune system [14]. They are highly expressed in sentinel cells such as macrophages and dendritic cells and recognized by structurally conserved molecules derived from microbes and activate immune cell responses [15]. One of the best-characterized TLRs is TLR2 by which macrophages recognize a wide range of ligands and mediates destructive chronic inflammatory reactions or confers host protection in acute infections [16]. However, most studies have focused on the function of the TLR2 gene, but its upstream regulator and the role of chromatin modification in this process have not been elucidated.

In the present study, we explored the role of CD38 in macrophage-mediated inflammatory response and the possible mechanisms. The data presented here showed that CD38 deficiency reduced the expressions of TLR2 at both mRNA and protein levels in CD38^−/−^ macrophages, and the mechanisms might be associated with activation of Sirt1/NF-*κ*B signaling. In addition, we observed that CD38 deficiency promoted the releases of some inflammatory cytokines in macrophages, indicating that CD38 deficiency promotes inflammatory response probably through Sirt1/NF-*κ*B-mediated inhibition of TLR2 expression in macrophages.

## 2. Materials and Methods

### 2.1. Cell and Reagents

RAW264.7 cells were maintained in RPMI 1640 media (GIBCO, Los Angeles, CA, USA) with 10% fetal bovine serum, 100 U/ml penicillin, and 100 *μ*g/ml streptomycin at 37°C with a humidified atmosphere containing 5% CO_2_. The pGL3 Luciferase Reporter Vectors and the Dual Luciferase Reporter assay system were obtained from Promega (Madison, WI, USA). The human dCas9 plasmid (no. 44247) and human sgRNA plasmid (no. 44248) were from Addgene. The overexpression plasmid pcDNA-CD38 was kindly provided by Dr. Xuan Huang in our lab. The antibodies against CD38, TLR2, NF-*κ*B p65, acetyl-NF-*κ*B p65, F4/80, CD11b, and GAPDH were obtained from Abcam (Cambridge, MA, USA). The acetylated histone 4 lysine 16 residue (H4 Ac-K16) antibody was from Cell Signaling Technology (Danvers, MA, USA). NAD, resveratrol, and nicotinamide were purchased from Sigma-Aldrich (St. Louis, MO, USA).

### 2.2. Isolation of Murine Peritoneal Macrophages

Peritoneal macrophages were isolated from CD38 knockout or wild-type mouse with C57BL/6J genetic background. The CD38^−/−^ mice were kindly provided by Dr. Frances E. Lund (University of Rochester, USA). Murine peritoneal macrophages were isolated as previously described [17]. Briefly, mice were injected with 1 ml of 3% Brewer thioglycollate medium into the peritoneal cavity, and inflammatory response was allowed to proceed for 4 days. Then, the animals were euthanized and a 20 G needle was inserted through a peritoneal wall along the mouse's left side and 5 ml of the cold harvest medium was injected into each mouse. Then, the fluid was aspirated from the peritoneum and was centrifuged in a refrigerated centrifuge for 10 min at 400 ×g at 4°C. The cells were resuspended in cold RPMI 1640 media and adjusted to 5 × 10^6^ cells/ml. The cells were placed at 37°C for 2 h, and no adherent cells were removed by gently washing with warm PBS. Macrophages were characterized using fluorescently labeled monoclonal antibodies F4/80 (at 1/20 dilution) and CD11b (at 1/50 dilution) by flow cytometry.

### 2.3. CD38 Knockdown in RAW264.7 Cells

CD38 knockdown experiments in RAW264.7 cells were performed using the CRISPR/Cas9 method as previously described [18]. Briefly, based on the sequences of the mouse CD38 gene, guide RNAs (sgRNAs) were selected to target the promoter region (MusCD38-up1 and MusCD38-up2) or exons (MusCD38-E1a, MusCD38-E1b, MusCD38-E1c, and MusCD38-E3) of CD38 and were ligated to human sgRNA plasmid. All sgRNA sequences targeting the mouse CD38 gene are summarized in Table S1 in the Supplementary Material. RAW264.7 cells were co-transfected with dCas9 plasmid (2 *μ*g) and human sgRNA plasmid (2 *μ*g) by electroporation (exponential wave, 500 *μ*F). The medium was changed after 12 h. Twenty-four hours later, puromycin (2 *μ*g/mL) was added. Cells were incubated at 37°C in a CO_2_ incubator for an additional 48 h prior to harvesting cells for mRNA and protein assay. The transfection with the dCas9 plasmid alone was performed as a negative control.

### 2.4. Real-Time PCR

Total RNA was isolated from the cultured macrophages using TRIzol reagent (Life Technologies, Grand Island, NY). One microliter of total RNA was reverse-transcribed using a 1st Strand cDNA Synthesis Kit with a thermocycler (GeneAmp PCR System 2720; Applied Biosystems, Foster City, CA). Real-time PCR was performed in the ABI 7900 sequence detection system (Applied Biosystems) with a reaction mixture that consisted of a SYBR Green 2× PCR Master Mix (Applied Biosystems), cDNA template, forward primer, and reverse primer. Primer sequences are as follows: 5′-TTTGCTGGAGCCCATTGAG-3′ and 5′-CATTATCTTGCGCAGTTTGCA-3′ (TLR2), and 5′-ACATGGCCTCCAAGGAGTAAGAA-3′ and 5′-GGGATAGGGCCTCTCTTGCT-3′ (GAPDH, for an endogenous control). The PCR protocol consisted of 40 cycles of denaturation at 95°C for 15 s followed by 60°C for 1 min to allow extension and amplification of the target sequence. Data were analyzed using the ABI 7900 sequence detection system software. The amount of TLR2 mRNA was normalized to GAPDH using the 2^−ΔΔCT^ method.

### 2.5. Western Blot Analysis

Protein levels were determined in homogenates of macrophages. Fifty micrograms of protein per sample was loaded in each lane and separated by SDS-PAGE and transferred to PVDF membranes in a Tris-glycine buffer (48 mM Tris, 39 mM glycine, pH 9.2) containing 20% methanol. The membranes were blocked with skimmed milk for 1 h, washed in Tris-buffered saline containing 0.1% Tween-20 (TBST), and incubated with primary antibodies overnight at 4°C. The dilution of primary antibodies was performed as follows: CD38 at 1/500 dilution, TLR2 at 1/200 dilution, NF-*κ*B p65 at 1/10000 dilution, acetyl-NF-*κ*B p65 at 1/2000 dilution, and GAPDH at 1/10000 dilution. The membranes were incubated for 1 h at room temperature with the horseradish peroxidase conjugated goat anti-rabbit IgG (1/5000 dilution; Santa Cruz Biotechnology Inc., CA, USA). The immunodetected protein bands were then detected using the ChemiDoc XRS system with Quantity One software (Bio-Rad, Richmond, CA, USA).

### 2.6. Bioinformatics Analysis and Luciferase Assays

The promoter region of TLR2 at the −5 kb position relative to the transcription start site was selected for bioinformatics analysis, using the online software Motifmap (http://motifmap.ics.uci.edu/). The luciferase reporter plasmids were derived from pGL3-Basic lacking the eukaryotic promoter sequences. A series of truncated TLR2 constructs were derived from the pGL3-TLR2 promoter by PCR. All of the constructs were confirmed by sequencing without coding frame shifts in the luciferase gene.

RAW264.7 cells were seeded in 24-well plates, grown to 50% confluence, and transfected with the constructs using Lipofectamine 2000 according to the manufacturer's instructions. To normalize the transfection efficiency, pRL-TK plasmids containing the Renilla luciferase gene were used as internal controls in each experiment. The activities of firefly and Renilla luciferases were determined from a single sample with the Dual Luciferase Reporter assay system using a luminometer, 48 h after transfection.

### 2.7. Mouse Cytokine Antibody Array

Assay for Mouse Cytokine Antibody Array (RayBiotech, cat. number AAM-INF-1) was carried out in accordance with the manufacturer's instructions. Briefly, antibody-coated array membranes were first incubated with 1 ml of blocking buffer. After 30 min, membranes were treated with the supernatant from WT, dCas9, CD38-E1a, and CD-38 E3 and allowed to incubate overnight at 4°C. The membranes were washed with a washing buffer and then incubated with 1 ml biotin-conjugated antibodies overnight at 4°C. Mixture of biotin-conjugated antibodies was removed, and membranes were incubated with HRP-conjugated streptavidin. Detection of spots using chemiluminescence was acquired with semiquantitative analysis of signal intensities from Quantity One software.

### 2.8. Statistical Analysis

Unless otherwise stated, all of the experiments were performed a minimum of three times. The results represent the mean ± S.D. from three experiments. We compared the data using one-way ANOVA or a *t*-test as appropriate and defined the statistical significance at *p* < 0.05.

## 3. Results

### 3.1. CD38 Deficiency Inhibits TLR2 Expression in Macrophages

**Figure 1:**
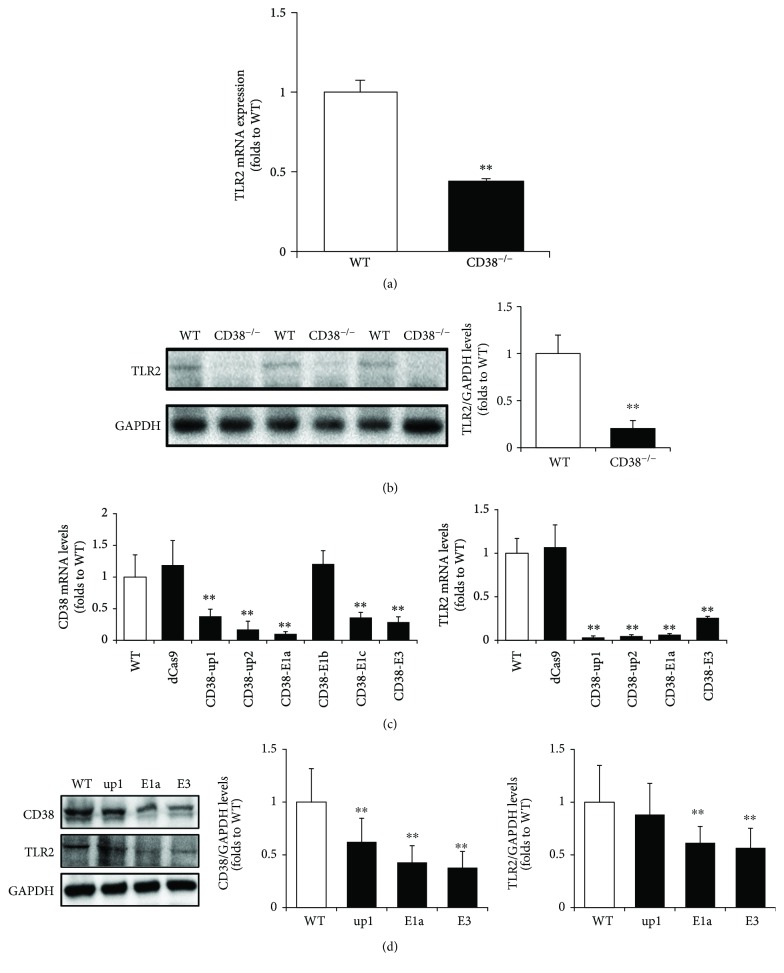
CD38 deficiency attenuates the expressions of TLR2 mRNA and protein in macrophages. The expressions of TLR2 mRNA (a) and protein (b) were quantitatively analyzed with primary macrophages from WT and CD38^−/−^ mice, and GAPDH was used as an internal control. The expressions of CD38 and TLR2 mRNA (c) and protein (d) were determined by qRT-PCR and Western blot, respectively, in RAW264.7 cells after a 72 h transfection with various CD38 sgRNAs. The data represent the mean ± S.D. from three independent experiments, ^∗∗^ *p* < 0.01 compared with the WT group.

We first examined the mRNA expression and protein levels of TLR2 in primary macrophages from WT and CD38^−/−^ mice. As shown in Figure 1(a), CD38 deficiency induced a significant downregulation of TLR2 mRNA levels, decreasing to 43.88 ± 2.41% compared with the WT macrophages (Figure 1(a)). Consistent with the mRNA result, the protein level of TLR2 was decreased to 20.78 ± 8.06% in CD38^−/−^ macrophages compared with the WT group (Figure 1(b)). We further verified the effects of CD38 deficiency on TLR2 expression in RAW264.7 macrophages. A total of six sgRNAs were designed and transfected into RAW264.7 cells. The blockade of different sites on the promoter region or exons caused a remarkable decrease in CD38 mRNA expression except CD38-E1b. In addition, TLR2 mRNA levels were reduced accordingly in CD38-up1, -up2, -E1a, and E3 groups (Figure 1(c)). Western blot results confirmed the knockdown of CD38 by CRISPRi/dCas9. The protein levels of CD38 were dropped to about 30% compared with WT in CD38-E1a and CD38-E3 groups. TLR2 levels were also decreased significantly in these two groups (Figure 1(d)). Therefore, the CD38-E1a and CD38-E3 cell line was used in our subsequent experiments.

### 3.2. NF-*κ*B Acts as the Potential Transcription Factor of TLR2 in Macrophages

**Figure 2:**
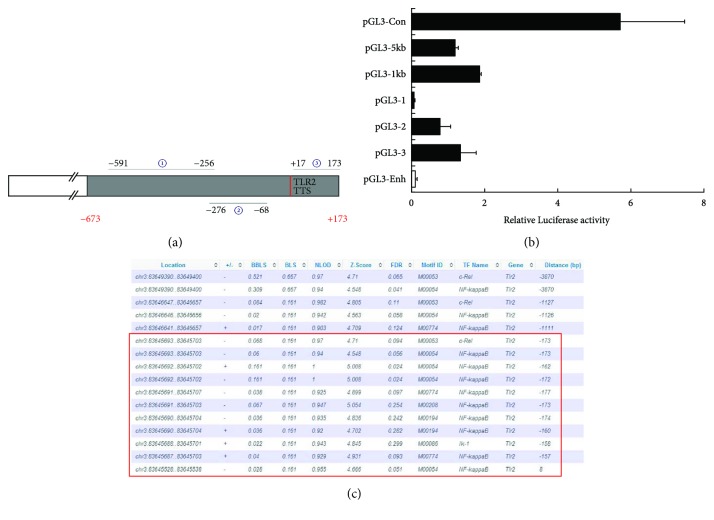
Identification of the transcription factors of the TLR2 gene in RAW264.7 cells. A series of truncated TLR2 promoter luciferase constructs were prepared, and the numbers represent the distance from the transcription start site to the truncated positions (a). The luciferase activities of the constructs of the various truncated TLR2 promoters were determined and normalized to the Renilla luciferase activity (b). The potential transcription factor-binding motifs of TLR2 were determined by using the Motifmap software programs (c). The data represent the mean ± S.D. from three independent experiments.

Since TLR2 protein and mRNA expression is markedly reduced in CD38^−/−^ macrophages, we proposed that CD38 may regulate the transcription of the TLR2 gene. Therefore, we subsequently analyzed transcriptional activity of the ~5 kb (−4102~1089) and ~1 kb (−673 to +173) regions of the TLR2 promoter by luciferase assays (Figure 2(a)). The cells were first transfected with the constructs pGL3-5kb and pGL3-1kb. As shown in Figure 2(b), these two plasmids had a comparative transcriptional activity, which implies that the possible *cis*-acting elements located within the 1 kb region may contribute to the transcription of the TLR2 gene. Therefore, we examined the transcriptional activity of three truncated constructs at the −591 to −256 (pGL3–1), −276 to −68 (pGL3–2), and + 17 to +173 (pGL3–3) positions, respectively (Figure 2(a)). Results showed that pGL3-2 and pGL3-3 produced significant luciferase activity (Figure 2(b)). We further identified the possible binding motifs of certain transcription factors by Motifmap software and found that the TLR2 promoter contained several binding motifs of NF-*κ*B at the −173 to 8 position (Figure 2(c)). These results indicate that NF-*κ*B may act as the potential transcription factor to specifically interact with the predicted binding site in the TLR2 promoter.

### 3.3. CD38 Deficiency Downregulates NF-*κ*B Levels in Macrophages

**Figure 3:**
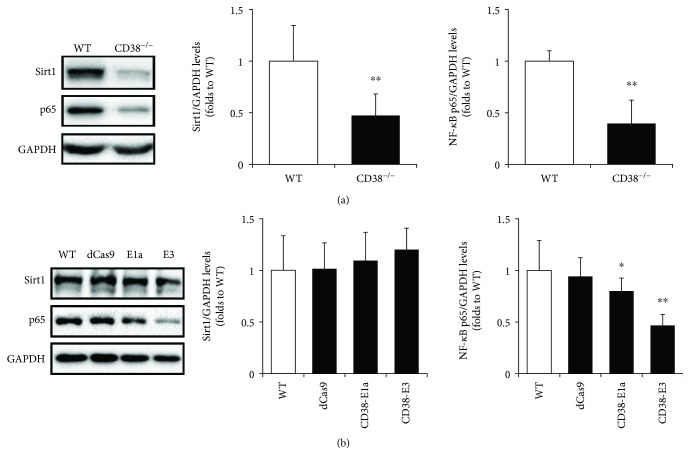
CD38 deficiency suppresses NF-*κ*B expression in macrophages. The protein levels of Sirt1 and NF-*κ*B p65 were determined by Western blot in primary macrophages (a) and RAW264.7 cells (b). The different clones of RAW264.7 cells, including the negative control dCas9 and the CD38 knockdown cell line E1a and E3, were selected for the experiment. The GAPDH was used as an internal control. The data represent the mean ± S.D. from three independent experiments, ^∗^ *p* < 0.05, ^∗∗^ *p* < 0.01 compared with the WT group.

Subsequently, we investigated whether CD38 has effects on Sirt1/NF-*κ*B signaling in macrophages. The data in Figure 3(a) showed that both the protein levels of Sirt1 and NF-*κ*B p65 in CD38^−/−^ macrophages were reduced to 47.24 ± 20.95% and 40.36 ± 21.57% compared with the WT group, respectively. Similarly, CD38 knockdown by E1a and E3 in RAW264.7 cells resulted in a downregulation of NF-*κ*B p65 but had no obvious effect on Sirt1 expression (Figure 3(b)).

### 3.4. Inhibition of Sirt1/NF-*κ*B Signaling Regulates TLR2 Expression in RAW264.7 Cells

**Figure 4:**
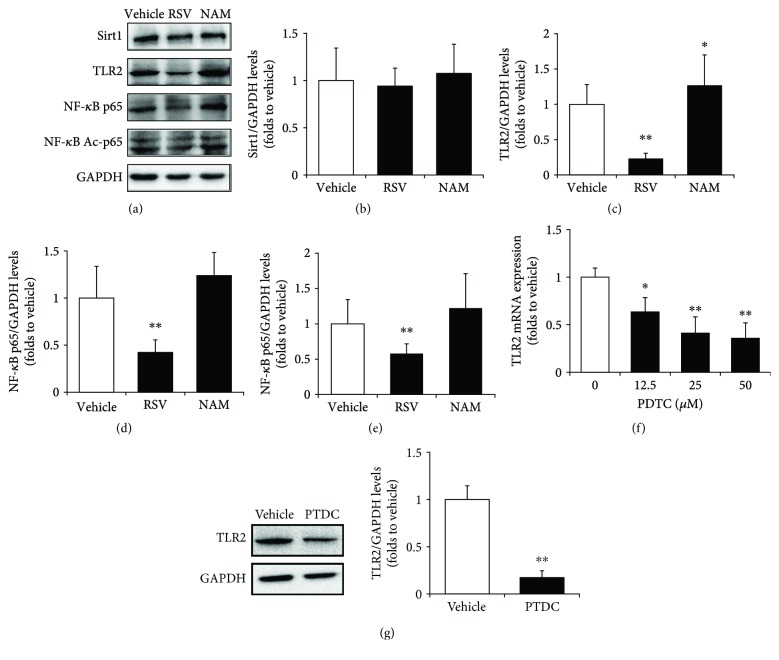
Sirt1/NF-*κ*B regulates TLR2 expression in RAW264.7 cells. The expressions of Sirt1, TLR2, NF-*κ*B p65, and acetyl-p65 (Ac-p65) proteins were detected by Western blot (a) and quantitatively determined for Sirt1 (b), TLR2 (c), NF-*κ*B p65 (d), and NF-*κ*B Ac-p65 (e) in RAW264.7 cells treated with Sirt1 antagonist resveratrol (RSV) or Sirt1 inhibitor nicotianamine (NAM). GAPDH was used as an internal control. The mRNA expressions of TLR2 were examined by qRT-PCR in RAW264.7 cells treated with various concentrations of NF-*κ*B inhibitor PDTC (f), and the protein levels of TLR2 were determined by Western blot treated with PDTC in RAW264.7 cells (g). The data represent the mean ± S.D. from three independent experiments, ^∗^ *p* < 0.05 and ^∗∗^ *p* < 0.01 compared with the vehicle group.

We applied resveratrol (RSV, a Sirt1 agonist), nicotinamide (NAM, a Sirt1 antagonist), and PDTC (an NF-*κ*B inhibitor) to verify whether Sirt1/NF-*κ*B signaling plays a role in the regulation of TLR2 expression in macrophages. RAW264.7 cells were treated with 20 *μ*M of RSV and 10 mM of NAM, respectively. After 24 h of incubation, cells were harvested for Western blot assay. Results showed that although Sirt1 levels did not alter in RSV- or NAM-treated groups compared with the vehicle-treated group (Figures 4(a) and 4(b)), TLR2 was significantly reduced to 23.3 ± 3.7% after RSV treatment and increased to approximately 27% with the treatment of NAM (Figures 4(a) and 4(c)). Similarly, p65 and Ac-p65 were decreased to 42.2 ± 13.4% and 57.8 ± 14%, respectively, in RSV-treated cells compared with control groups. In addition, NAM induced a slight increase in p65 and Ac-p65 levels, but there were no significant differences (Figures 4(a), 4(d), and 4(e)).

RAW264.7 cells were first treated with various concentrations of PDTC for 12 hours, and then the total RNA was extracted for real-time PCR assay. The results showed that TLR2 expressions were decreased by PDTC treatment in a dose-dependent manner (Figure 4(f)). PDTC (25 *μ*M) resulted in an obvious decrease in TLR2 protein levels in RAW264.7 cells with a reduction to 17.38 ± 7.18% compared with the vehicle group (Figure 4(g)). These results suggest that Sirt1/NF-*κ*B signaling regulates TLR2 expression in macrophages.

### 3.5. Re-Expression of CD38 Reverses the Effects on Sirt1/NF-*κ*B/TLR2 Signaling in CD38-Deficient RAW264.7 Cells

**Figure 5:**
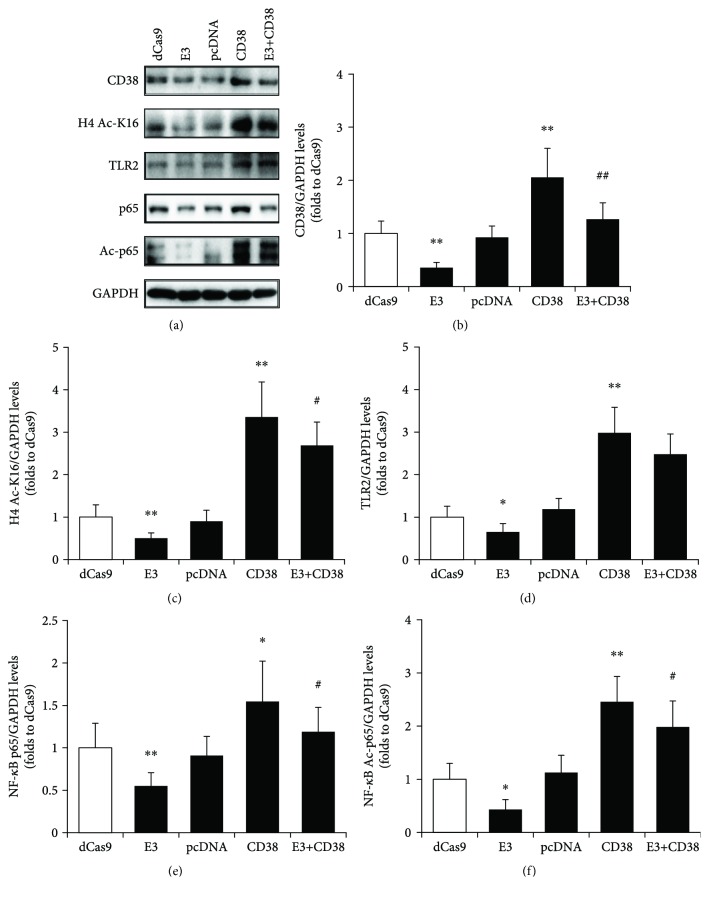
Re-expression of CD38 in RAW264.7 cells reverses Sirt1/NF-*κ*B/TLR2 signaling. The different clones of RAW264.7 cells, including the negative control dCas9 and pcDNA, the CD38 knockdown cell line E3, the overexpression clone of CD38, and the line co-transfected with E3 and CD38 (E3+CD38), were selected for the experiment. The expressions of CD38, acetylated histone 4 lysine 16 residue (H4 Ac-K16), TLR2, NF-*κ*B p65, and acetyl-p65 (Ac-p65) proteins were detected by Western blot (a) and quantitatively determined for CD38 (b), H4 Ac-K16 (c), TLR2 (d), NF-*κ*B p65 (e), and NF-*κ*B Ac-p65 (f). The levels of H4 Ac-K16 represent the activity of Sirt1. GAPDH was used as an internal control. The data represent the mean ± S.D. from three independent experiments. ^∗^ *p* < 0.05 and ^∗∗^ *p* < 0.01 compared with the dCas9 group; ^#^ *p* < 0.05 and ^##^ *p* < 0.01 compared with the CD38 group.

To verify that the activation of the Sirt1/NF-*κ*B/TLR2 signaling pathway was through CD38, an overexpression plasmid of CD38 was transfected into WT or CD38 knockdown (CD38-E3) RAW264.7 cells. The transfection with the dCas9 or pcDNA plasmid was set as the negative control. We assessed Sirt1 activity in RAW264.7 using an antibody specific for acetylated histone 4 lysine 16 residues (H4 Ac-K16). CD38 knockdown significantly decreased H4 Ac-K16 immunoreactivity, indicating an increased Sirt1 activity in RAW264.7 cells. CD38 overexpression induced an increased level of H4 Ac-K16, which was strongly blocked by re-expression of CD38 in the knockdown cell lines (Figures 5(a) and 5(b)). The downstream TLR2, NF-*κ*B p65, and Ac-p65 were also detected. As expected, re-expression of CD38 reverses the effects on NF-*κ*B/TLR2 signaling in CD38-deficient RAW264.7 cells (Figures 5(a), 5(c)–5(f)). These results confirmed that CD38 deficiency affected Sirt1 activity thus blocking NF-*κ*B/TLR2 signaling in macrophage.

### 3.6. CD38-Mediated Sirt1/NF-*κ*B/TLR2 Signaling Is NAD-Dependent

**Figure 6:**
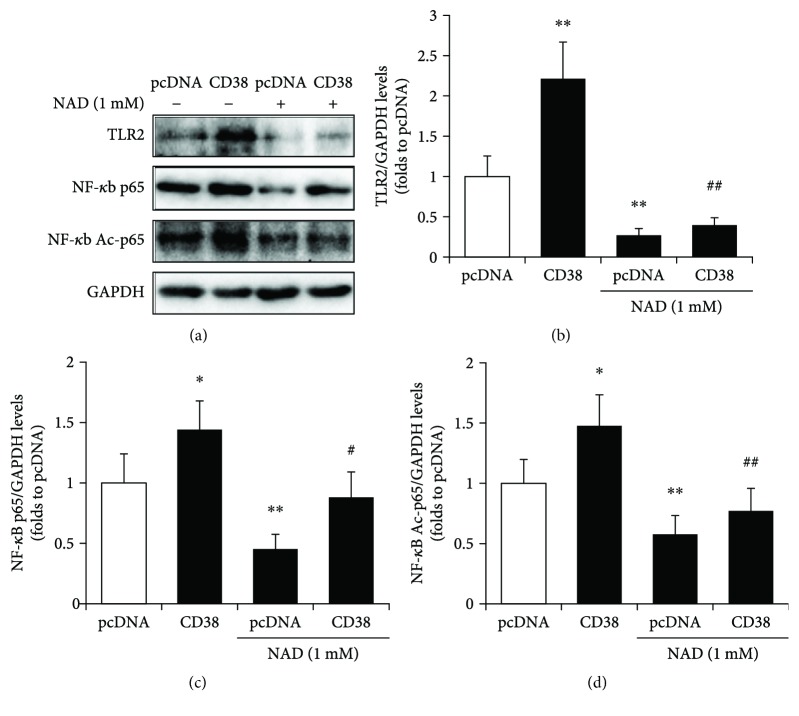
CD38-mediated Sirt1/NF-*κ*B/TLR2 signaling is NAD-dependent. The expressions of TLR2, NF-*κ*B p65, and acetyl-p65 (Ac-p65) proteins were detected by Western blot (a) and quantitatively determined for TLR2 (b), NF-*κ*B p65 (c) and NF-*κ*B Ac-p65 (d) in control and CD38-overexpressed RAW264.7 cells in the absence or presence of NAD. GAPDH was used as an internal control. The data represent the mean ± S.D. from three independent experiments. ^∗^ *p* < 0.05 and ^∗∗^ *p* < 0.01 compared with the dCas9 group; ^#^ *p* < 0.05 and ^##^ *p* < 0.01 compared with the CD38 group.

Since CD38 is an NADase which controls Sirt1 activity, we further investigated the effects of NAD on Sirt1/NF-*κ*B/TLR2 signaling. The control and CD38 overexpression RAW264.7 cells were treated with 1 mM NAD for 24 h, and the protein levels of TLR2, NF-*κ*B p65, and Ac-p65 were detected. CD38 induced a remarkable increase in TLR2 levels, as well as the levels of p65 and Ac-p65. However, NAD treatment decreased the expression of TLR2, p65, and Ac-p65 (Figures 6(a)–6(d)). These results suggested that CD38 mediated Sirt1/NF-*κ*B/TLR2 signaling in an NAD-dependent manner.

### 3.7. CD38 Deficiency Promotes the Release of Proinflammatory Molecules in RAW264.7 Cells

**Figure 7:**
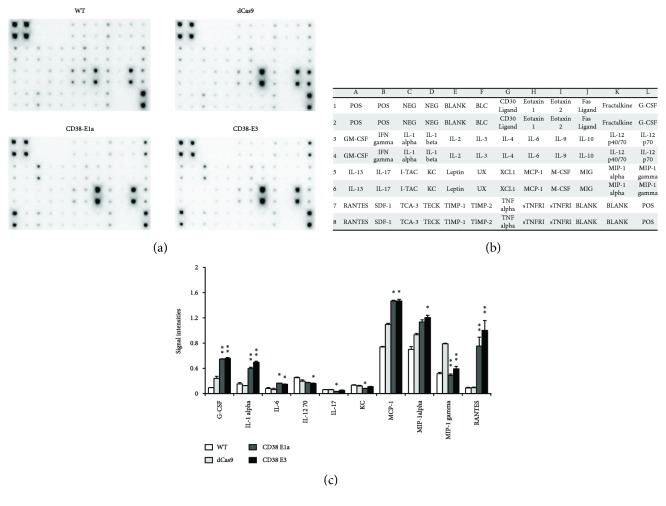
Chemokine secretions are regulated by CD38 in macrophages. An expression profile of chemokine proteins was obtained by performing a chemokine antibody array with WT, the negative control dCas9, and the CD38 knockdown clones E1a and E3 RAW264.7 cells (a). Template showing the location of chemokine antibodies spotted onto the RayBio chemokine array (b). Mean optic densities of protein were calculated by normalizing to positive controls, and results were compared (c). The data represent the mean ± S.D. from three independent experiments. ^∗^ *p* < 0.05 and ^∗∗^ *p* < 0.01 compared with the dCas9 group.

In order to observe the profile of cytokine production in control and CD38 deficiency (CD38-E1a and CD38-E3) macrophages, a RayBio® murine cytokine array was utilized to assess the quantity of proinflammatory molecules. Relative expression levels of 40 chemokines were determined by densitometry (Figure 7(b)). Compared with the dCas9 group, a total of 10 chemokines were changed significantly. Knockdown of CD38 promoted the production of G-CSF, IL-1alpha, IL-6, MCP-1, and RANTES in both knockdown cell lines, while only MIP-1 gamma was attenuated in CD38-E1a and CD38-E3 cells. The levels of IL-12 70, IL-17, and KC were reduced in one of the cell lines (Figures 7(a) and 7(c)). On the whole, CD38 deficiency resulted in the proinflammatory phenotype.

## 4. Discussion

The present study shows that CD38 plays a crucial role in TLR2 transcription and protein expression in macrophages. We identified several NF-*κ*B-binding motifs in the 5-flanking region of the promoter of the TLR2 gene. Moreover, our data suggest that CD38 deficiency significantly induced downregulation of TLR2, at least partially, through deacetylation of NF-*κ*B p65 by Sirt1. The elevated proinflammatory cytokine production in CD38 knockdown cells may be attributed to the decreased TLR2 levels.

Recent studies have demonstrated that CD38 plays an important role in inflammatory-related diseases. CD38^−/−^ mice display an ameliorated inflammatory response in arthritis and infection models [9, 19–21]. However, the proinflammatory effects in CD38-deficient models were also reported. Matalonga et al. [22] demonstrated that LXR agonists protect host macrophages from extensive bacterial infection through transcriptional activation of CD38 and subsequent reduction in intracellular NAD levels. These results were consistent with our proinflammatory phenotype in macrophages. The contradictory conclusion might depend on the cell type used in the experiment. However, there is little evidence showing the mechanisms of CD38-mediated inflammation in macrophage.

TLRs play important roles in the initiation of innate immune responses against invading microorganisms and are involved in the recognition of a wide range of ligands such as nucleic acids, lipids, lipoproteins, and polysaccharides [23]. Changes in TLR ligand binding and signaling capacity will result in changed innate immunity and contribute to increased susceptibility to a myriad of infectious diseases [24, 25] and to some noninfectious diseases [26, 27]. Accordingly, we initially examined the expression profile of TLRs in macrophages and found that CD38-deficient macrophages from mice induced a significant decrease in TLR2 at both mRNA and protein levels. We proposed that there may be a certain upstream regulator involved in this process. Thus, a 5 kb region of the upstream sequences in the promoter of the mouse TLR2 gene was examined and a 1 kb region with significant luciferase activity was identified. We further used motif analysis to search for any transcription factors that might be involved in the regulation of TLR2 expression in macrophages and found that there are several binding motifs of NF-*κ*B at the −173 to 8 position. These results indicated that NF-*κ*B may be the potential transcription factor in the regulation of TLR2 expression.

Sirt1 is an NAD^+^-dependent deacetylase that has been shown to deacetylate histone H4 lysine residue 16 (H4K16) as well as a number of nonhistone targets including p53, Ku70, PPAR*γ*, PGC-1*α*, NF-*κ*B, HIF-2*α*, XPA, and several FOXO isoforms [28]. Yeung et al. demonstrated that Sirt1 could directly interact with and deacetylate the RelA/p65 component of the NF-*κ*B complex [29]. The deacetylation of Lys310 inhibited the transactivation capacity of the RelA/p65 subunit and consequently suppressed the transcription of the NF-*κ*B-dependent gene expression [30, 31]. CD38 is a major regulator of the cellular/nuclear NAD^+^ level and Sirt1 activity. In macrophage, CD38 mediates the increase in NADase activity and the reduction in intracellular NAD levels, while increased NAD levels were observed after CD38 deficiency [22]. In the present study, we investigated whether reduced TLR2 levels are associated with Sirt1 or NF-*κ*B activity. We confirmed the increased activity of Sirt1 in CD38 knockdown RAW264.7 cells by detecting the acetylation status of H4K16 [32]. Not in consistency with its increased activity, the protein levels of Sirt1 were reduced in CD38^−/−^ macrophages, suggesting that there might be a negative feedback regulation. We also found that NF-*κ*B p65 levels were decreased, which may be regulated by the activation of Sirt1 in CD38^−/−^macrophages.

To further explore the molecular basis for Sirt1/NF-*κ*B-mediated regulation of the TLR2 gene, certain agonists and inhibitors involved in this pathway were introduced. We demonstrated that activation of Sirt1 attenuated the acetylation of p65 and downregulated TLR2 levels. Inhibition of NF-*κ*B also resulted in the decreased expression of TLR2, suggesting that CD38 deficiency-mediated deacetylation of NF-*κ*B resulted in a downregulation of TLR2 in macrophages. In addition, re-expression of CD38 reverses the effects on NF-*κ*B/TLR2 signaling in CD38-deficient RAW264.7 cells. These results further confirmed that TLR2 expression is regulated by NF-*κ*B acetylation via deacetylase Sirt1 in macrophages. Furthermore, we observed the effect of NAD on Sirt1/NF-*κ*B signaling in control and CD38-overexpressing cells. The activation of the NF-*κ*B/TLR2 signaling pathway induced by CD38 was reversed by the addition of exogenous NAD treatment, which suggested that CD38 modulates the Sirt1/NF-*κ*B signaling pathway in an NAD-dependent manner.

Finally, we performed the Cytokine Antibody Array assay to provide information on the cytokine secretion profile in CD38 deficiency macrophages. Of the 40 cytokines determined in this system, 5 cytokines including G-CSF, IL-1alpha, IL-6, MCP-1, and RANTES were significantly increased in CD38-E1a and CD38-E3 cells, and MIP-1alpha was increased only in CD38-E3 cells. G-CSF, a glycoprotein, stimulates the bone marrow to produce granulocytes and stem cells. G-CSF promoted the proliferation of neutropenic progenitor cells which were differentiated into granulocytes and accelerated the release of neutrophils from the PMC into the blood [33]. IL-1 alpha, MCP-1, and IL-6 are important proinflammatory cytokines, which induce the adhesion, migration, and infiltration of immune cells such as neutrophils and monocytes/macrophages and exaggerate inflammatory response [34–36]. RANTES and MIP-1 alpha are members of the CC chemokine family. RANTES is chemotactic for T cells, eosinophils, and basophils and plays an active role in recruiting leukocytes into inflammatory sites. MIP-1 alpha activates human granulocytes which can lead to acute neutrophilic inflammation. It also induces the synthesis and release of other proinflammatory cytokines such as IL-1, IL-6, and TNF-*α* from fibroblasts and macrophages [37]. Four chemokines were found to be reduced in one of the knockdown cell lines, and only MIP-1 gamma obviously attenuated in both knockdown cells. It is not clear why CD38 knockdown exhibited a downregulation in the proinflammatory mediator MIP-1 gamma and needs further investigation in our laboratory. In general, CD38 deficiency promoted the production of proinflammatory cytokines in macrophages.

It is noted that TLR2 has recently been recognized as capable of eliciting anti-inflammatory responses [38, 39]. The heterodimerization of TLR2 has been considered a factor potentially determining the ensuing pro- versus anti-inflammatory responses. In general, TLR2/1 complexes have been more often linked with proinflammatory responses than TLR2/6 complexes, which have been linked with anti-inflammatory responses [40]. However, the precise role of TLR2 in the regulation of pro- or anti-inflammatory signaling remains unclear at the moment. In this study, we observed the increased proinflammatory cytokines accompanied with the downregulation of TLR2 expression in CD38^−/−^ macrophages, suggesting that CD38-mediated inflammatory response may be partly attributed to the altered TLR2 levels. However, whether CD38 deficiency contributes to a proinflammatory phenotype and whether TLR2 expression is regulated by the Sirt-1/NF-*κ*B signaling pathway in primary macrophages remain unknown, and whether the decreased TLR2 expression in CD38-deficient macrophages under normal conditions is also observed following an exogenous stimulus by the TLR2-specific agonists is still not clear but warrants further investigation.

**Figure 8:**
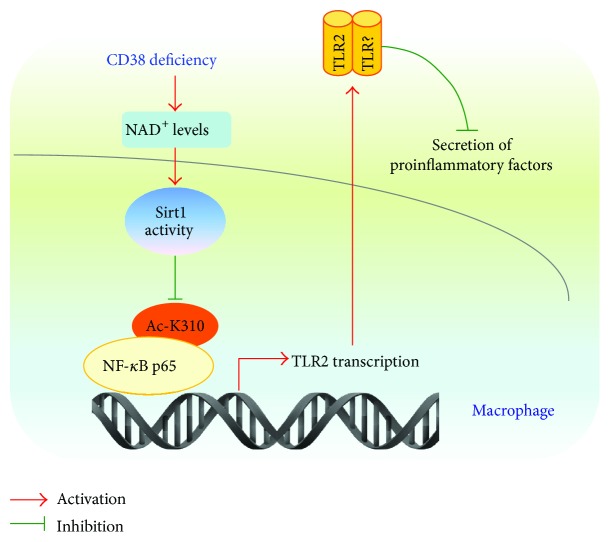
A schematic diagram of the mechanism by which CD38 deficiency suppresses TLR2 expression and promotes chemokine secretions.

Taken together, the results from this study indicate that CD38 deficiency increases NAD^+^ levels and Sirt1 activity, which promotes NF-*κ*B deacetylation in the promoter region of the TLR2 gene and subsequently inhibits the transcription of TLR2 (Figure 8). Our findings may provide a novel insight into the molecular mechanisms underlying CD38-mediated inflammatory response in macrophages, which might play a role in the setting of infectious disease.

## Data Availability

The raw/processed data required to reproduce these findings cannot be shared at this time due to technical or time limitations.

## Supplementary Materials

Supplementary Materials Table S1: sgRNA sequences targeting the mouse CD38 gene.
