# Supplementary material for: CD38 Deficiency Promotes Inflammatory Response through Activating Sirt1/NF-κB-Mediated Inhibition of TLR2 Expression in Macrophages
Source: Mediators Inflamm. 2018 May 27;2018:8736949. doi: 10.1155/2018/8736949 (PMC6011090; doi:10.1155/2018/8736949)
Supplement: Supplementary Materials — Table S1: sgRNA sequences targeting the mouse CD38 gene. [file 8736949.f1.pdf]

Mouse CD38 gene knockdown was performed using CRISPR interference (CRISPRi). Different sites upstream (MusCD38-up1and MusCD38-up2) or in the exons (MusCD38-E1a, MusCD38-E1b, MusCD38-E1c and MusCD38-E3) of CD38 gene were selected and ligated to human sgRNA plasmid to block transcription initiation or elongation. All sgRNA sequences target to mouse CD38 gene were summarized in Tab. S1.

Tab. S1. sgRNA sequences target to mouse CD38 gene

Name	Sequences
MusCD38-up1	GGAGAACCACCTTGTTGGGGGCTCCCGAAGACTGGCCCGTTTTAGAGCT<br>AGAAATAGCAAGTTAAAATAAGGC
MusCD38-up2	GGAGAACCACCTTGTTGGGAACTTTGGAGCAGGAGTAGGTTTTAGAGCT<br>AGAAATAGCAAGTTAAAATAAGGC
MusCD38-E1a	GGAGAACCACCTTGTTGGGCCGCGCTCACTCCTGGTGTTTTAGAGCTAG<br>AAATAGCAAGTTAAAATAAGGC
MusCD38-E1b	GGAGAACCACCTTGTTGGGGACCCACTCCGAGACCGATCGTTTTAGAGC<br>TAGAAATAGCAAGTTAAAATAAGGC
MusCD38-E1c	GGAGAACCACCTTGTTGGGACCACGATCCCTACTACCAGTTTTAGAGCT<br>AGAAATAGCAAGTTAAAATAAGGC
MusCD38-E3	GGAGAACCACCTTGTTGGGCTGATGATCTCAGGTGGTGGTTTTAGAGCT<br>AGAAATAGCAAGTTAAAATAAGGC
sgRNA R	CCTAGTACTCGAGAAAAAAAGCACCGACTCGGTGCCACTTTTTCAAGTT<br>GATAACGGACTAGCCTTATTTTAACTTGCTATTTCTAGCTCTAAAAC

The sgRNA-targeting sequences are underlined and are indicated in red.
